# MiR-199a Inhibits Secondary Envelopment of Herpes Simplex Virus-1 Through the Downregulation of Cdc42-specific GTPase Activating Protein Localized in Golgi Apparatus

**DOI:** 10.1038/s41598-017-06754-3

**Published:** 2017-07-27

**Authors:** Kyousuke Kobayashi, Fumiko Suemasa, Hiroshi Sagara, Shinya Nakamura, Yasushi Ino, Kazuyoshi Kobayashi, Hiroaki Hiramatsu, Takeshi Haraguchi, Kazuo Kurokawa, Tomoki Todo, Akihiko Nakano, Hideo Iba

**Affiliations:** 1 Division of Host-Parasite Interaction, Department of Microbiology and Immunology, Institute of Medical Science, University of Tokyo, Tokyo, 1088639 Japan; 2 Fine Morphological Analysis Group, Medical Proteomics Laboratory, Institute of Medical Science, University of Tokyo, Tokyo, 1088639 Japan; 3 Division of Innovative Cancer Therapy, Advanced Clinical Research Center, Institute of Medical Science, University of Tokyo, Tokyo, 1088639 Japan; 4 Laboratory of Developmental Cell Biology, Department of Biological Science, Graduate School of Science, University of Tokyo, Tokyo, 1130033 Japan; 5 Live Cell Super-Resolution Imaging Research Team, Extreme Photonics Research Group, RIKEN Center for Advanced Photonics, Saitama, 3510198 Japan; 6 Division of RNA Therapy, Medical Mycology Research Center, Chiba University, Chiba, 2608673 Japan

## Abstract

Because several studies have shown that exogenous miR-199a has antiviral effects against various viruses, including herpesviruses, we examined how miR-199a exerts its antiviral effects using epithelial tumour cell lines infected with herpes simplex virus-1 (HSV-1). We found that both miR-199a-5p and -3p impair the secondary envelopment of HSV-1 by suppressing their common target, ARHGAP21, a Golgi-localized GTPase-activating protein for Cdc42. We further found that the *trans*-cisternae of the Golgi apparatus are a potential membrane compartment for secondary envelopment. Exogenous expression of either pre-miR-199a or sh-ARHGAP21 exhibited shared phenotypes i.e. alteration of Golgi function in uninfected cells, inhibition of HSV-1 secondary envelopment, and reduction of *trans*-Golgi proteins upon HSV-1 infection. A constitutively active form of Cdc42 also inhibited HSV-1 secondary envelopment. Endogenous levels of miR-199a in epithelial tumour cell lines were negatively correlated with the efficiency of HSV-1 secondary envelopment within these cells. These results suggest that miR-199a is a crucial regulator of Cdc42 activity on Golgi membranes, which is important for the maintenance of Golgi function and for the secondary envelopment of HSV-1 upon its infection.

## Introduction

The host gene regulatory network is a strong determinant of viral susceptibility because viral replication is highly dependent on cellular proteins and can also be restricted by antiviral host factors. MicroRNAs (miRNAs) are a class of small non-coding RNA that post-transcriptionally regulate genes by binding to partially complementary sequences in the 3′ untranslated region (3′UTR) of target mRNAs. Given the importance of cellular miRNAs in diverse biological processes, including development, differentiation, homeostasis, and the stress-response, and in diseases such as cancer and fibrosis, some miRNAs would be expected to have inhibiting or enhancing effects upon viral replication by forming cellular gene regulatory networks. However, the significance of this system is still poorly understood.

The *miR-199a/miR-214* (*miR-199a-2*) locus, from which the transcript is processed into miR-199a-5p, miR-199a-3p, and miR-214, is a good model for studying the function of host miRNAs in viral replication because several previous studies have described the antiviral effects of miR-199a-3p and miR-214 against herpesviruses, Semliki Forest virus^1^, hepatitis C virus (HCV)^2^, and hepatitis B virus^3^, raising the possibility that these miRNAs target host factor(s) that are critically involved in the replication of many types of virus.

A growing body of evidence has indicated the importance of miR-199a-5p and miR-199a-3p, both produced from a single molecule of pre-miR-199a, in development, differentiation, the response to hypoxia, endoplasmic reticulum (ER) stress and tumorigenesis^4–7^, via the targeting of many cellular factors. Our previous studies have revealed that miR-199a-5p and miR-199a-3p form gene regulation networks in human epithelial tumour cell lines by suppressing their common target, Brm, a catalytic subunit of the SWI/SNF complex, and further that these tumour cell lines tend to fall into either of two steady states: miR-199a(−)/Brm(+) and miR-199a(+)/Brm(−) through a miR-199a/Brm/EGR1 axis^8, 9^.

To better understand the mechanisms by which these miRNAs inhibit viral replication, we have here used epithelial tumour cell lines infected with herpes simplex virus-1 (HSV-1), a member of the α-herpesvirus subfamily, as a model system. HSV-1 infects human epithelial cells from skin lesions or mucosae. By fusion of the HSV-1 envelope with the endosomal or plasma membrane, the HSV-1 capsid is internalized into the host cytoplasm and transported to the nuclear membrane where it injects double-stranded genomic DNA into the nuclei. Through a cascade of HSV-1 gene expression phases (immediate early genes, early genes, and late genes), genomic DNA is replicated and packaged into capsids in the nucleus. These capsids are enveloped at the inner nuclear membrane and then bud into the space between the inner and outer nuclear membrane, a process known as primary envelopment. This envelope is fused with the outer nuclear membrane to deliver a capsid into the cytoplasm. Capsids acquire tegument proteins on their way to cytoplasmic membrane compartments for secondary envelopment. Mature, infectious virions inside the compartments are transported to the extracellular space^10, 11^.

In the present study, we show that both miR-199a-5p and miR-199a-3p suppress the secondary envelopment of HSV-1, which largely occurs at the *trans*-region of the Golgi apparatus. These miRNAs were further found to target ARHGAP21, a GTPase-activating protein (GAP) for Cdc42. We additionally show that appropriate regulation of Cdc42 at the Golgi apparatus is required for the maintenance of the membrane compartments of HSV-1 secondary envelopment.

## Results

### Both miR-199a-5p and -3p disturb HSV-1 replication in various human cell lines

**Figure 1:**
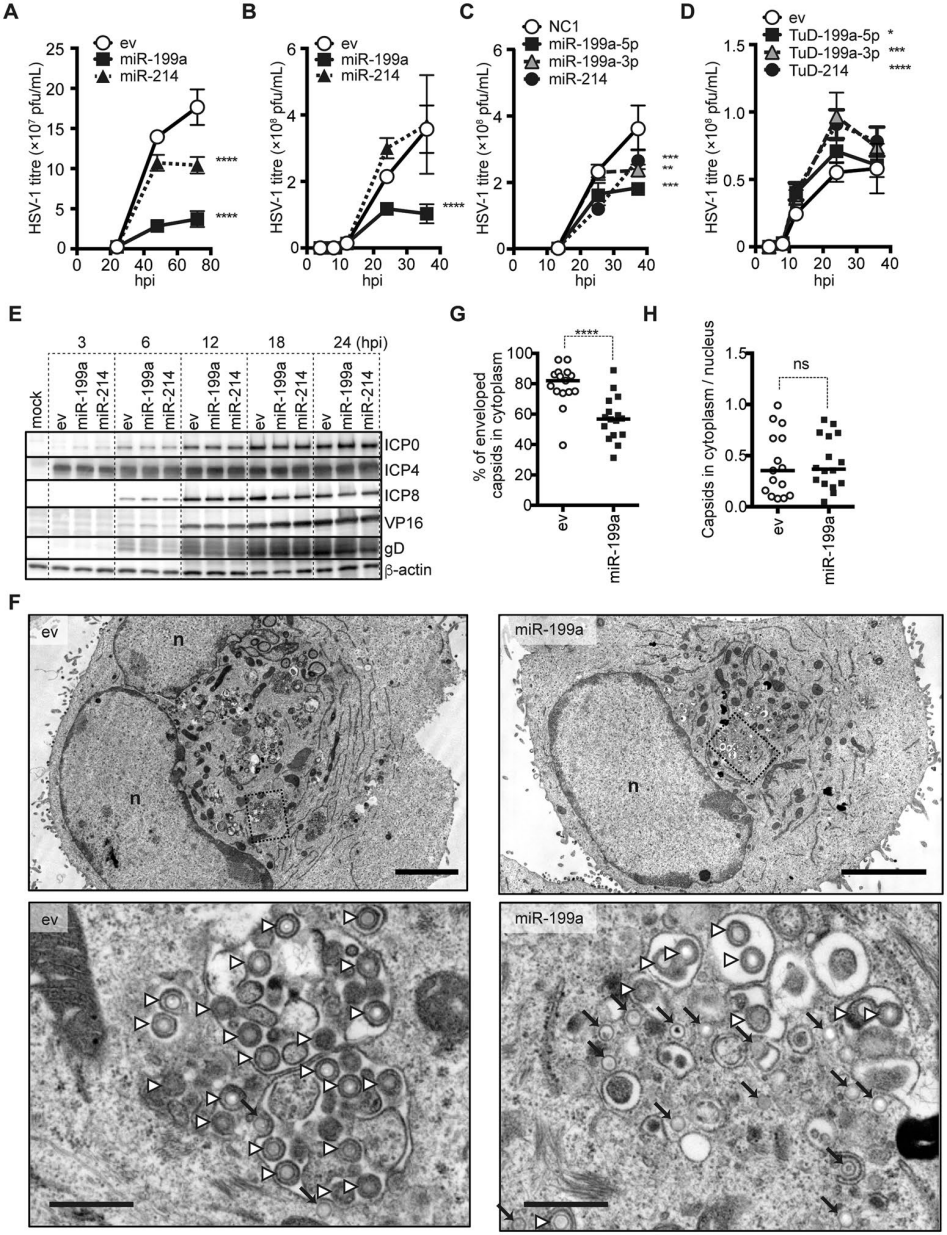
Both miR-199a-5p and miR-199a-3p disturb HSV-1 replication by inhibiting HSV-1 secondary envelopment. (**A**–**C**) A549 cells transduced with control (ev) or pre-miRNA-expressing lentivirus vector (**A** and **B**) or transfected with an miRNA mimic (**C**) were infected with HSV-1 at an moi of 0.01 (**A**) or 5 (**B** and **C**). Virus samples were collected at 4, 8, 12, 24, 36, 48, and/or 72 hpi (hours post infection). HSV-1 titres in the cell culture supernatant were determined by plaque assay. (**D**) HUTU80 cells transduced with control (ev) and TuD-expressing lentivirus vector were infected with HSV-1 at an moi of 5. Virus samples were collected at 4, 8, 12, 24, and/or 36 hpi. HSV-1 titres in the cell culture supernatant were determined by plaque assay. (**E**) Western blot analysis of immediate early gene products (ICP0 and ICP4), the early gene product (ICP8), late gene products (VP16 and gD), and β-actin protein in HSV-1-infected A549 cells (moi of 5), which were transduced with control (ev) or pre-miRNA-expressing lentivirus vector. (**F**) Representative transmission electron micrographs of HSV-1-infected A549 cells (moi of 5/20 hpi), which were transfected with control (ev) or pre-miR-199a-expressing lentivirus vector. The lower panels (bars indicate 500 nm) show magnifications of the areas boxed with dashed lines in the upper panels (bars indicate 4 µm). The white arrowheads indicate enveloped capsids and the arrows indicate non-enveloped capsids in cytoplasm. (**G**) Percentages of enveloped capsids versus total capsids in the cytoplasm, calculated by counting capsids in TEM images. (**H**) Ratios of HSV-1 capsids (either enveloped or not) per unit area in the cytoplasm to those in the nucleus, calculated by counting capsids in TEM images. The data in (**A–D**) represent the means ± s.d. (n = 3). In (**G** and **H**), the bars represent the medians. The asterisks indicate the *p* value compared with ev (two-way ANOVA in (**A–D**) and *t*-test in **G** and **H**). ns, not significant. *p < 0.05, **p < 0.01, ***p < 0.005, ****p < 0.001.

A previous study reported that miR-199a-3p and miR-214 inhibit HSV-1 replication in a murine fibroblast cell line^1^. In our present investigation, we first tested the inhibitory effects of miR-199a and -214 on HSV-1 replication in human cell lines originating from epithelial tumours (Fig. 1A,B and Supplementary Fig. S1). In A549 cells, which express marginal levels of endogenous miR-199a/214^8^, the exogenous expression of pre-miR-199a via a lentivirus vector impaired HSV-1 replication at both a low and high multiplicity of infection (moi) of HSV-1, whereas miR-214 introduction through a pre-miR-214 vector marginally but significantly suppressed replication only at a low moi. In all of the other four cell lines tested, the pre-miR-199a vector produced a stronger suppression of HSV-1 than the miR-214 vector. Interestingly, the decreased replication of HSV-1 induced by pre-miR-199a was even observed in an interferon receptor-deficient cell line, HEC-1B^12^, suggesting that this effect is independent of the interferon-mediated antiviral pathway.

Because mature miR-199a-5p and -3p are simultaneously generated in the experiments using the pre-miR-199a vector, we next used miRNA mimics for the exogenous expression of each of these three miRNAs. In these experiments, miR-199a-5p, -3p, and -214 alone each impaired HSV-1 replication in A549 cells (Fig. 1C). To specifically inhibit these three miRNAs, we used the Tough Decoy (TuD) method that we previously developed^13^. Lentivirus vector expressing TuD against miR-199a-5p, -3p, or -214 (TuD-199a-5p, TuD-199a-3p, or TuD-214 vector, respectively) was transduced into HUTU80 cells, which endogenously express these miRNAs at high levels^8^. HSV-1 was found to be replicated in these cells more efficiently than in cells transduced with empty vector (Fig. 1D). These results suggest that both miR-199a-5p and -3p inhibit efficient HSV-1 replication in various human cell lines, whereas the inhibitory effect of miR-214 is cell line dependent.

### MiR-199a primarily impairs the secondary envelopment step or steps immediately before the secondary envelopment

To identify the point of the infection cycle that is inhibited by miR-199a, we examined the time course of HSV-1 gene expression (Fig. 1E and Supplementary Fig. S1). Control and miRNA-transduced cells showed very similar expression levels of the immediate early genes, early genes, and late genes, indicating that miR-199a targets are not required for the cascade of virus gene expression and suggesting that this miRNA inhibits the later phase of viral replication, including capsid assembly, nuclear egress, secondary envelopment, and exocytosis of infective particles. We next observed intracellular capsids by transmission electron microscopy (TEM) (Fig. 1F). In TEM images, we can detect two distinct capsids, which would most certainly represent enveloped capsids (white arrowheads) and naked capsids (black arrows), respectively; The majority of capsids shown by white arrowheads were enclosed within compartments away from the nucleus, supporting that the secondary envelopment at the time of fixation is detectable by TEM under our observation conditions. Therefore, we used the ratios of enveloped capsids to total capsids in the cytoplasm to estimate the efficiency of secondary envelopment. These ratios were much lower in cells transduced with the pre-miR-199a vector than in those transduced with empty vector (Fig. 1G). Importantly, the ratios of total capsids in the cytoplasm to capsids in the nucleus were almost independent of exogenous miR-199a expression (Fig. 1H).

To assess the efficiency of virion secretion after secondary envelopment, we separately collected cell-free (culture supernatants) and cell-associated virus and determine the ratio of cell-free virus (Supplementary Fig. S1). Regardless of the presence of miR-199a, infective HSV-1 virions were secreted into the culture supernatant at very similar ratios, suggesting that miR-199a does not inhibit the secretion of HSV-1 virion. These results suggested that miR-199a primarily impairs the secondary envelopment step or steps immediately before the secondary envelopment, including assembly of tegument on cytoplasmic capsids and transport of envelope proteins to the site of secondary envelopment of HSV-1.

### The *trans*-region of the Golgi apparatus is a potential membrane compartment for secondary envelopment in A549 cells

**Figure 2:**
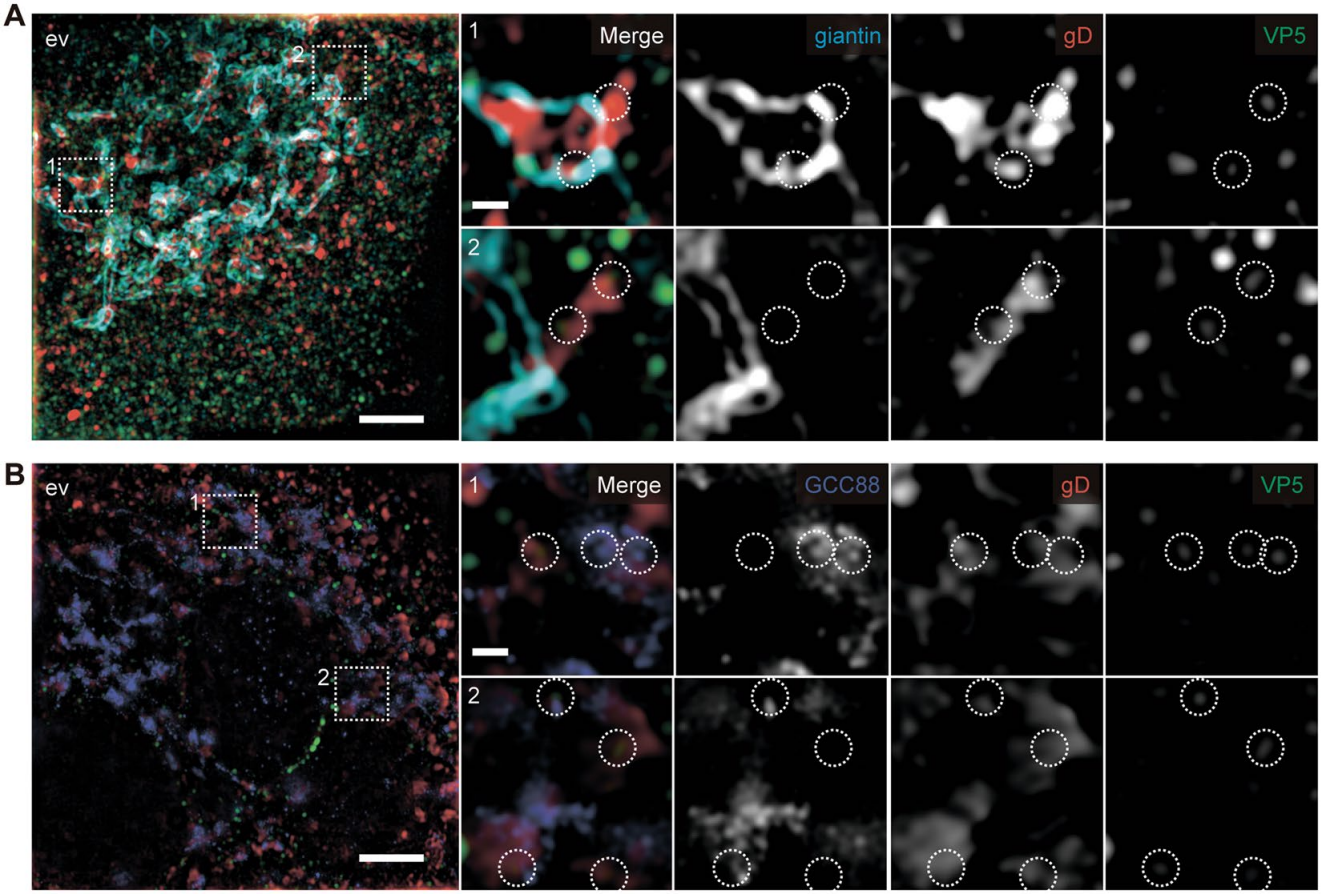
The *trans*-cisternae of the Golgi apparatus are a potential membrane compartment for secondary envelopment. (**A** and **B**) Representative super-resolution images of HSV-1-infected A549 cells (moi of 5/12 hpi) transfected with control (ev) lentivirus vector. Cells were simultaneously stained with anti-gD antibody (red) and anti-VP5 antibody (green) in combination with anti-giantin antibody (**A**, cyan) or anti-GCC88 antibody (**B**, blue). The farthest left panels show z-stack images reconstructed by maximum intensity projection (bars, 5 µm). The other panels show magnifications of the two areas boxed with dashed lines (1,2) in the left panels (bars, 1 µm). The circles indicate capsids associated with or included in gD-positive membrane compartments.

We here focused on secondary envelopment and host factors involved in this step. Although the origin of the cytoplasmic membrane compartment used for secondary envelopment is still debated, the *trans*-Golgi network (TGN)^14, 15^, multivesicular bodies^16^, and endocytic compartments^17, 18^ have all been reported as potential sites for the secondary envelopment of HSV-1. We used super-resolution microscopy to determine the secondary envelopment site in A549 cells. VP5 staining mostly showed distinct punctate signals with a diameter of approximately 100 nm (capsids) in cytoplasm at 12 hpi (Fig. 2A,B), consistent with the previous reports that capsid proteins accumulate in cellular nuclei for capsid formation within 8 hpi and then begin to translocate to cytoplasm through primary envelopment at 7–8 hpi^19–21^. On the other hand, an envelope protein gD was detected exclusively in cytoplasm as larger indistinct areas, which would mainly indicate the transition steps of gD to the membrane compartment for secondary envelopment including translation in ER and glycosylation in the Golgi apparatus (Fig. 2A,B). Like several previous approaches to detect the site for secondary envelopment of this virus^21, 22^, we assumed that the capsid protein (VP5) puncta with approximately 100 nm in diameter that are associated with or included in the envelope protein (gD) areas are the possible sites for secondary envelopment. Indeed, we often detected these sites in close proximity to giantin (a medial- and *cis*-Golgi marker)-positive compartments (Fig. 2A), but sometimes more than 200 nm apart. In contrast to the findings of some earlier reports^21–24^, TGN46 (a TGN marker) was not found to reside in the envelopment sites (Supplementary Fig. S2), consistent with more recent reports^17, 18^.

We therefore next tested the possibility that the *trans* region of the Golgi apparatus (*trans*-Golgi) is the membrane compartment for secondary envelopment. Two *trans*-Golgi markers, p230 and GCC88, were detected with similar subcellular localizations that were clearly distinct from that of TGN46 (Supplementary Fig. S2). We detected gD-positive compartments associated with or including VP5 signals in GCC88-positive compartments (Fig. 2B). These data indicated that the membrane compartment in A549 cells for the secondary envelopment of HSV-1 includes the *trans*-Golgi.

### ARHGAP21 is a common target of both miR-199a-5p and -3p

**Figure 3:**
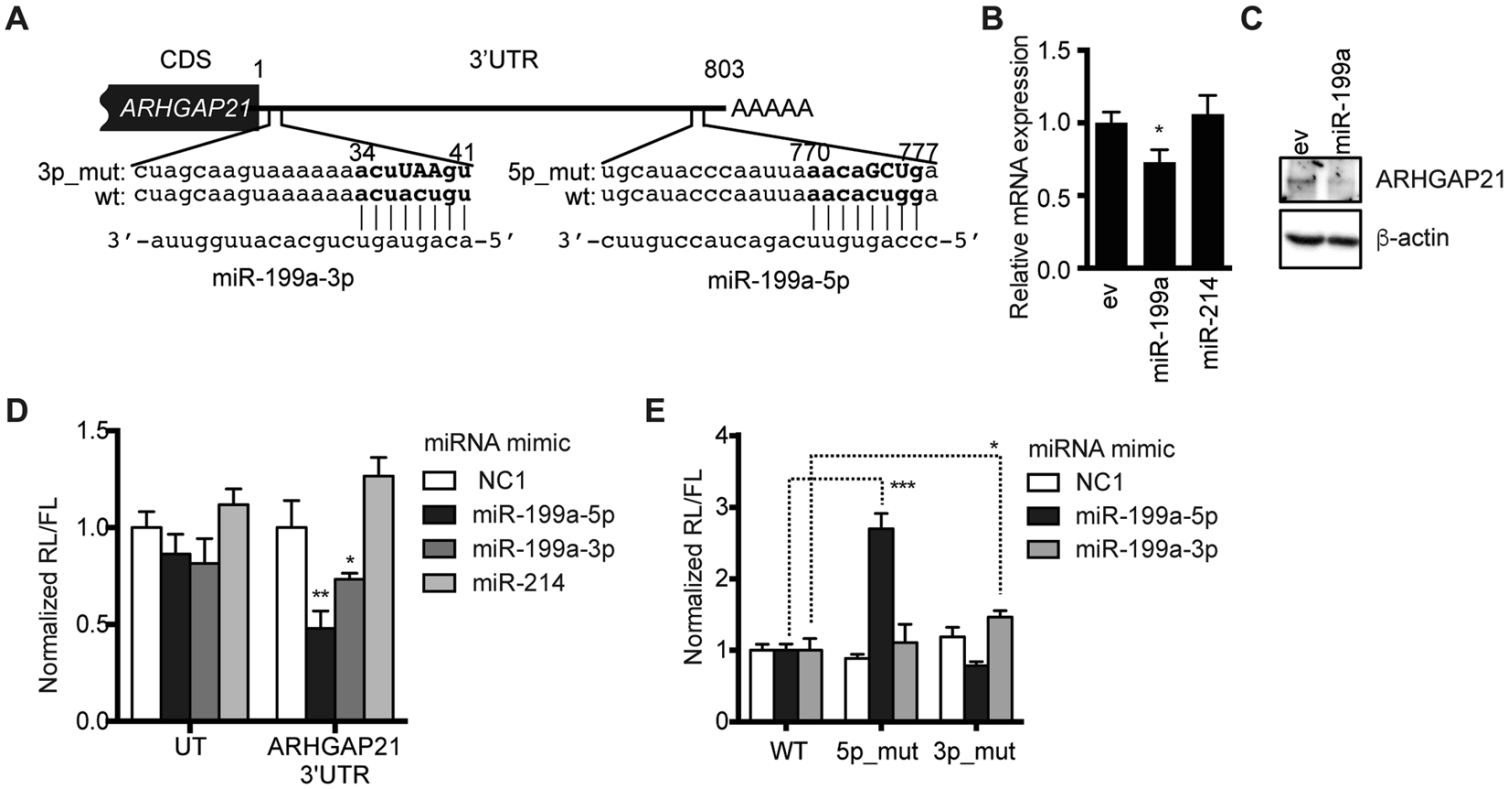
ARHGAP21 is a target of both miR-199a-5p and -3p. (**A**) Schematic diagram of the binding sites of miR-199a-5p and -3p within the 3′UTR of *ARHGAP21* mRNA (wt) and mutated sequences (3p_mut and 5p_mut). Sequences of seed sites are indicated in boldface and mutated bases in uppercase. (**B**) qRT-PCR analysis of *ARHGAP21* mRNA in A549 cells transduced with control (ev) or pre-miRNA-expressing lentiviral vector. GAPDH was used as an internal control. (**C**) Western blot analysis of ARHGAP21 and β-actin in A549 cells transduced with control or miR-199a-expressing lentiviral vector. (**D**) Expression of firefly *luciferase* genes containing the 3′UTR of *ARHGAP21* mRNA or an untargeted (UT) sequence, measured in the presence of an miRNA mimic. (E) Expression of firefly *luciferase* genes containing the 3′UTR of *ARHGAP21* mRNA (WT) or its mutants (5p_mut or 3p_mut), whose sequences are shown in (**A**), measured in the presence of an miRNA mimic. In (**B–E**), the data represent the means ± s.d. (*n* = 3). Asterisks indicate the *p* value (*t*-test) compared with the control (EV in **A** and NC1 in **D**) or with the wild-type 3′UTR of *ARHGAP21* (**E**). *p < 0.05, **p < 0.01, ***p < 0.005.

We hypothesized from our observations that crucial targets of miR-199a for the inhibition of HSV-1 secondary envelopment would reside around the Golgi apparatus. We thus prepared a putative list of gene targets of miR-199a-5p or -3p using target prediction algorithms from which we selected genes to which gene ontology (GO) terms associated with the Golgi apparatus can be assigned (Table 1). Of the 21 selected candidate genes, we focused on ARHGAP21 because it was predicted to be a good target of both miR-199a-5p and -3p (Fig. 3A) and because it has been reported to be important for Golgi function; ARHGAP21 is a Cdc42-specific GAP that can bind to ARF1, which reportedly recruits ARHGAP21 to the Golgi apparatus^25, 26^.

**Table 1:** Target identification using target prediction algorithms.

Gene ID	Accession Number	Target prediction			
		miR-199a-5p		miR-199a-3p	
		TargetScan^1^	PicTar^2^	TargetScan	PicTar
ABCA1	NM_005502	○		○	○
AFTPH	NM_001002243	○	○		
AP1G1	NM_001030007,NM_001128	○	○		○
ARHGAP21	NM_020824	○	○		○
B3GNT1	NM_006876	○	○		
CAV2	NM_001233,NM_001206747			○	○
CCDC88A	NM_018084,NM_001135597			○	○
CDC42	NM_044472			○	○
FN1	NM_002026			○	○
GANAB	NM_198335,NM_198334	○	○		
HSPA5	NM_005347	○	○		
LARGE	NM_004737	○	○		
MINK1	NM_015716,NM_001024937	○	○		
MTOR	NM_004958			○	○
NAA25	NM_024953			○	○
PDE4D	NM_001104631,NM_006203	○	○		○
ST6GAL1	NM_003032	○	○		
SULF1	NM_001128204,NM_015170	○	○		
TGIF2	NM_001199513,NM_021809			○	○
TMED5	NM_001167830,NM_016040			○	○
UBAP1	NM_016525,NM_001171201	○	○		

^1^ http://www.targetscan.org/. ^2^ http://pictar.mdc-berlin.de/.

In support of this target prediction, both *ARHGAP21* mRNA and protein expression were reduced by the exogenous stable expression of pre-miR-199a (Fig. 3B and C). The *luciferase* gene containing the 3′UTR of *ARHGAP21* mRNA was specifically downregulated by the miR-199a-5p and -3p mimics in the reporter plasmid (Fig. 3D). Importantly, this inhibition was reduced by the introduction of nucleotide changes into the predicted seed-binding sequences of miR-199a-5p and -3p (Fig. 3A and E). These data clearly indicated that both of these miRNAs directly target *ARHGAP21* transcripts.

### ARHGAP21 is important for HSV-1 secondary envelopment

**Figure 4:**
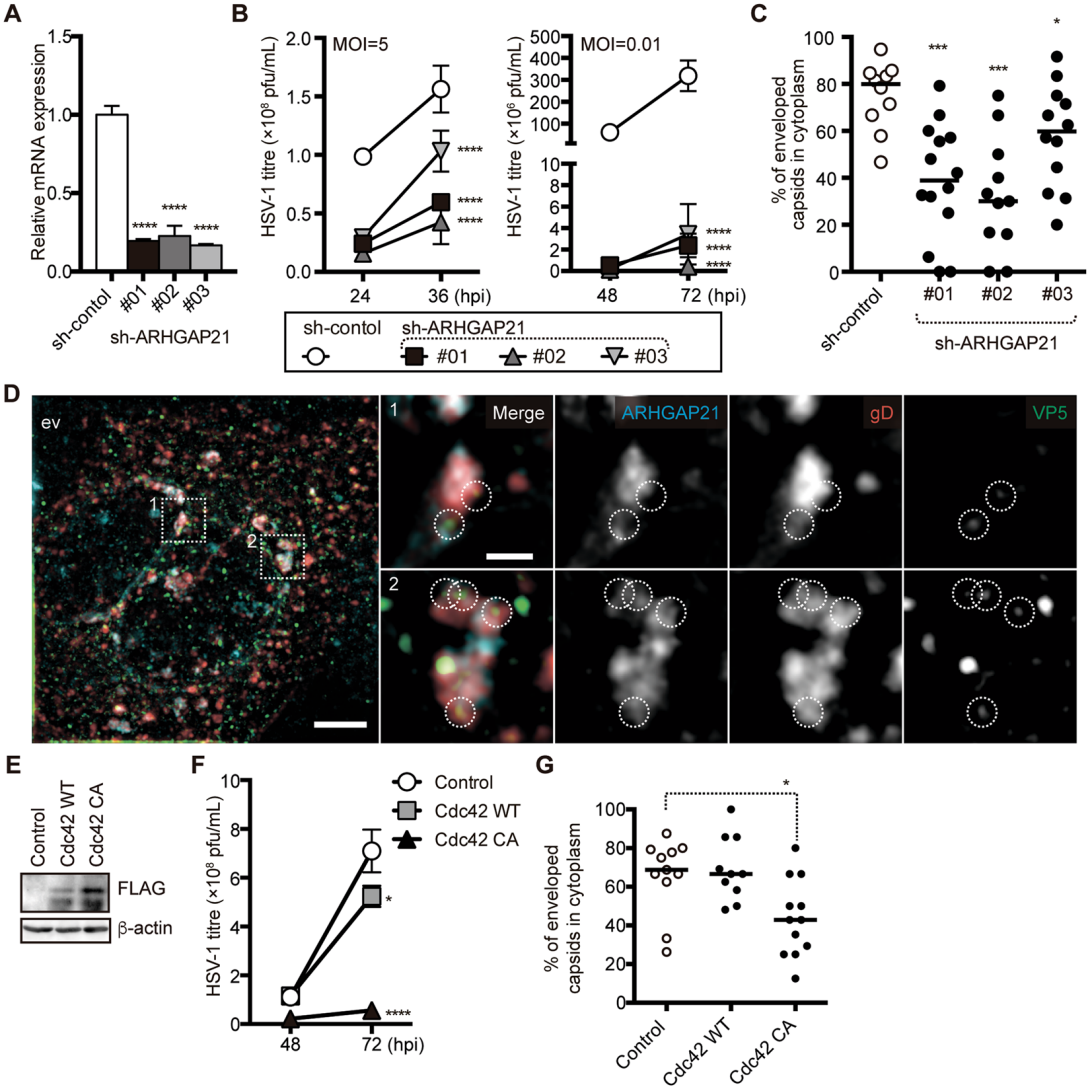
The ARF1-ARHGAP21-Cdc42 pathway is a crucial regulator of HSV-1 replication. (**A**) qRT-PCR analysis of *ARHGAP21* mRNA in A549 cells transduced with sh-control- or sh-ARHGAP21-expressing lentivirus vector. GAPDH was used as an internal control. (**B** and **F**) A549 cells transfected with sh-control and sh-ARHGAP21-expressing lentivirus vector (**B**) or GFP- (control), Cdc42 WT-, and Cdc42 CA-expressing retrovirus vector (**F**) were infected with HSV-1 (moi of 5). Titres in the cell supernatant of HSV-1-infected cells were determined by plaque assay at the times indicated. (**C** and **G**) Percentages of enveloped capsids versus total capsids in the cytoplasm, calculated by counting capsids in TEM images of HSV-1-infected A549 cells transfected with control and sh-ARHGAP21-expressing lentivirus vector (**C**) or with GFP- (control) and Cdc42 CA-expressing retrovirus vector (**G**). (**D**) Representative super-resolution images of HSV-1-infected A549 cells (moi of 5/12 hpi) transfected with control (ev) lentivirus vector. Cells were simultaneously stained with anti-gD antibody (red), anti-VP5 antibody (green), and anti-ARHGAP21 antibody (cyan). The furthest left panel shows z-stack images reconstructed by maximum intensity projection (bars, 5 µm) and the other panels show magnifications of the two areas boxed with dashed lines in the left panel (bars, 1 µm). The circles indicate capsids associated with or included in gD-positive membrane compartments. (**E**) Western blot analysis of FLAG-fusion proteins and β-actin in A549 cells transduced with GFP- (control), Cdc42 WT-, and Cdc42 CA-expressing retrovirus vectors. In (**A,B**, and **F**), the data represent the means ± s.d. (*n* = 3). In (**C** and **G)**, the bars represent the medians. In (**A–C**,**F**, and **G**), the asterisks indicate the *p* value (*t*-test in (**A**,**C**, and **G)** and two-way ANOVA in **B** and **F**) compared with sh-control (**A**–**C**) or GFP (control) (**F** and **G**). *p < 0.05, ***p < 0.005, ****p < 0.001. (291/350).

To evaluate the contribution of ARHGAP21 to HSV-1 replication, we designed three shRNAs, which efficiently repressed the expression of *ARHGAP21* mRNA (Fig. 4A) and strongly inhibited HSV-1 replication (Fig. 4B). TEM analysis also revealed that the efficient secondary envelopment of HSV-1 was disrupted in these ARHGAP21-depleted cells (Fig. 4C). These data thus indicated that ARHGAP21 is one of the key factors involved in the secondary envelopment of HSV-1. In support of this, we found that ARHGAP21 is abundant at gD- and VP5- positive areas, supporting the possibility that ARHGAP21 resides in the site of the secondary envelopment (Fig. 4D).

It has been reported that Cdc42 is a major effector protein of ARHGAP21 in the Golgi apparatus^25^. We thus generated a constitutively active (CA) mutant of Cdc42 to mimic Cdc42 activation (Fig. 4E) and thereby evaluate the role of Cdc42 in HSV-1 replication. In A549 cells expressing the Cdc42 CA mutant, HSV-1 replication was significantly decreased (Fig. 4F) and these cells showed similar phenotypes to cells expressing pre-miR-199a or sh-ARHGAP21 as judged by reduction in the percentage of enveloped capsids in the cytoplasm (Fig. 4G). Whereas the activation of Cdc42 CA mutant would not be specific to the Golgi apparatus, these observations are consistent with the idea that HSV-1 secondary envelopment, as well as steps immediately preceding the secondary envelopment, would be the key target point of Cdc42 activation.

### Endogenous expression levels of miR-199a-5p and -3p negatively correlate with the efficiency of HSV-1 secondary envelopment

**Figure 5:**
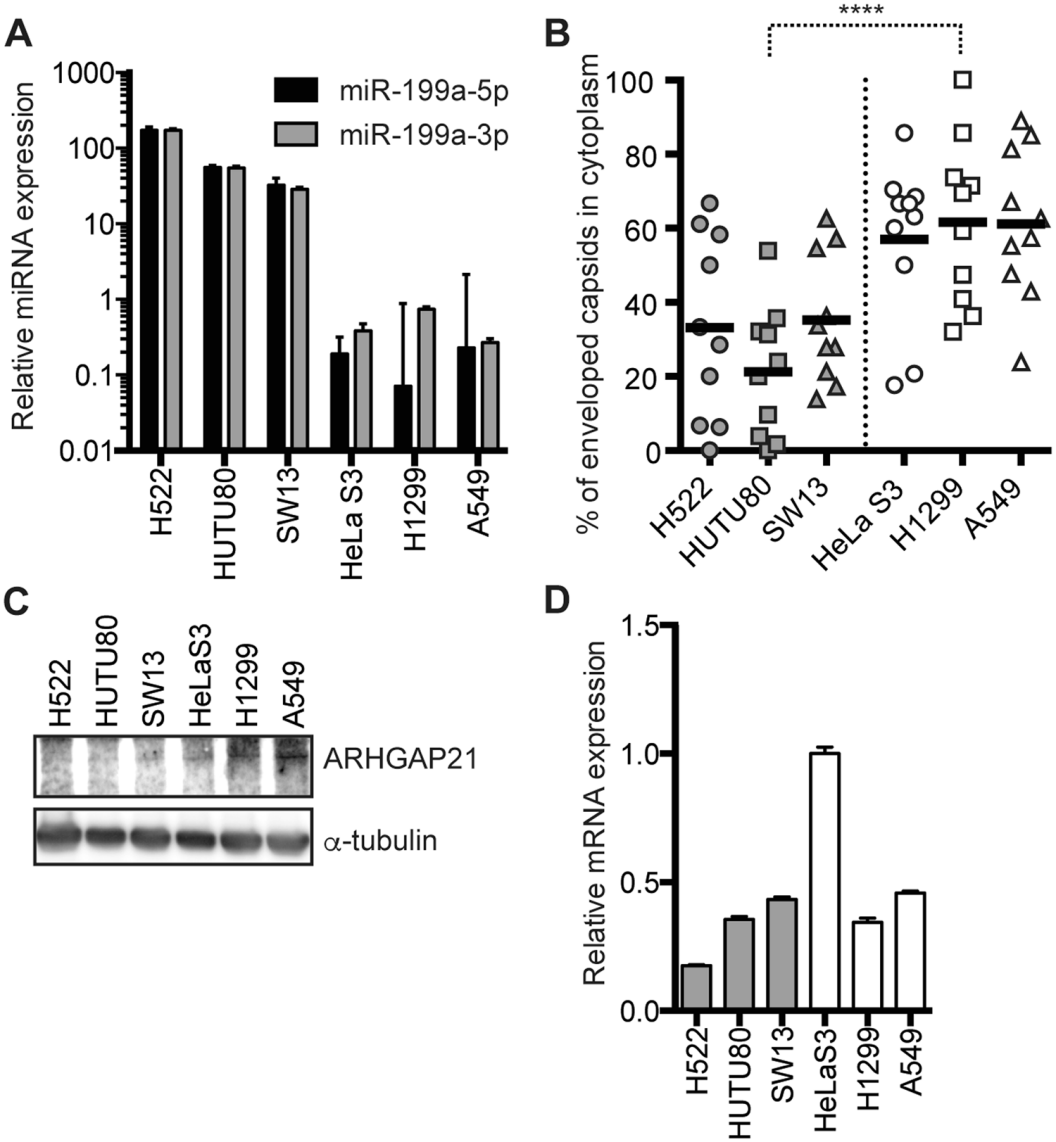
Endogenous expression levels of miR-199a-5p and -3p negatively correlate with secondary envelopment efficiency. (**A** and **D**) qRT-PCR analysis of miR-199a-5p and miR-199a-3p (**A**) and *ARHGAP21* mRNA (**D**) in human epithelial cancer cell lines. RNUB6 and GAPDH were used as an internal control for miRNA and mRNA quantification, respectively. (**B**) Percentages of enveloped capsids in the cytoplasm versus total capsids, calculated by counting capsids in TEM images of HSV-1-infected (moi of 5/20 hpi) human epithelial cancer cell lines. (**C**) Western blot analysis of ARHGAP21 and α-tubulin in human epithelial cancer cell lines. In (**A** and **D**), the data represent the means ± s.d. (*n* = 3). In B, the bars represent the medians and the asterisk indicates the *p* value (Mann-Whitney *U*-test). ****p < 0.001. (125/350).

We previously reported that human cell lines originating from epithelial tumours tend to fall into either of two steady states, miR-199a(−)/Brm(+) or miR-199a(+)/Brm(−), denoted type 1 cells and type 2 cells, respectively^8, 9^ (Fig. 5A). To examine the effects of endogenous miR-199a on HSV-1 infection, we determined the efficiency of HSV-1 secondary envelopment in these cell lines. The three type 1 cell lines showed a higher efficiency of the secondary envelopment than the three type 2 cell lines (Fig. 5B), indicating that the endogenous expression levels of miR-199a negatively correlate with the efficiency of HSV-1 secondary envelopment. ARHGAP21 protein was also at higher levels in the three type 1 cell lines (Fig. 5C) but the difference in *ARHGAP21* mRNA expression was less clear (Fig. 5D), possibly reflecting potent post-transcriptional suppression of *ARHGAP21* mRNA by both miR-199a-5p and miR-199a-3p. These data suggest that miR-199a endogenously regulates HSV-1 secondary envelopment via the downregulation of ARHGAP21 and that this regulation may occur in cell lines other than A549.

### Exogenous expression of miR-199a or sh-ARHGAP21 alters Golgi function in A549 cells

Several previous studies have suggested that activated Cdc42 specifically binds to the γ subunit of the COPI complex (γ-COP) in the Golgi apparatus^27^ and inhibits Golgi-to-ER and intra-Golgi retrograde transport mediated by COPI vesicles^27–29^, raising the possibility that miR-199a naturally modulates COPI transport through Cdc42 activation.

**Figure 6:**
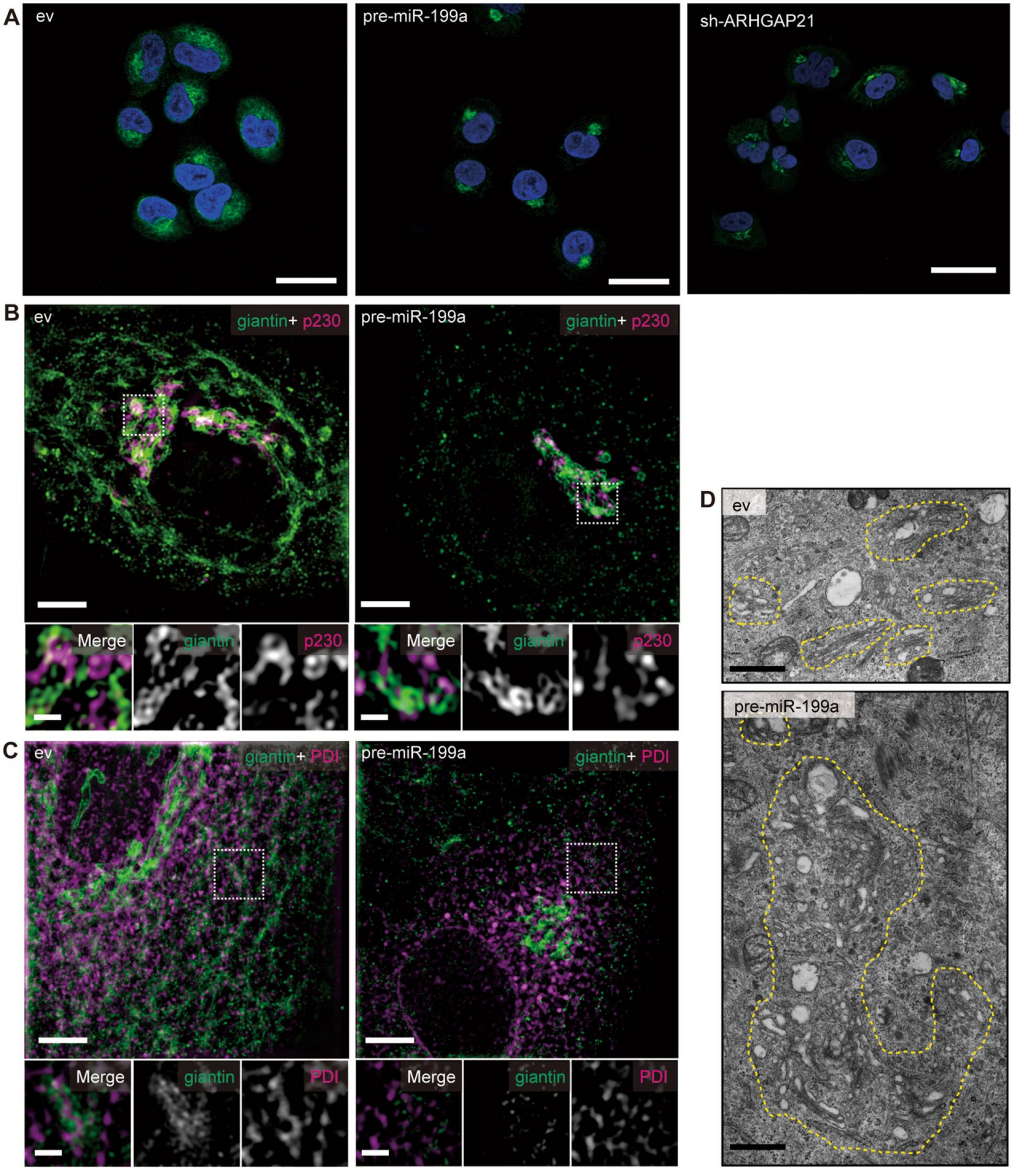
Exogenous expression of miR-199a or sh-ARHGAP21 in A549 cells alters the Golgi structure and function. (**A**) Representative confocal microscopy images of A549 cells transduced with control (ev), pre-miR-199a-, or sh-ARHGAP21#01-expressing lentivirus vector. Cells were stained with anti-giantin antibody (green) and DAPI (blue). In sh-ARHGAP21-expressing cells, the arrowhead indicates a cell with condensed distribution of giantin, and the arrow indicates a cell with meshwork distribution of giantin. Magnifications are shown in the lower panels (bars, 5 µm). (**B** and **C**) Representative super-resolution images of A549 cells transfected with control (ev) or pre-miR-199a-expressing lentivirus vector stained with anti-giantin antibody (green) in combination with anti-p230 antibody (**B**, magenta) or anti-PDI antibody (**C**, magenta). The upper panels show z-stack images reconstructed by maximum intensity projection (bars, 5 µm) and the lower panels show magnifications of the areas boxed with dashed lines in the upper panels (bars, 1 µm). (**D**) Representative transmission electron micrographs of A549 cells transfected with control (ev) or pre-miR-199a-expressing lentivirus vector (bars, 1 µm). Areas enclosed by dashed yellow lines indicate the Golgi apparatus.

To examine the effects of miR-199a on the Golgi apparatus, we analyzed the subcellular distribution of the medial- and *cis*-Golgi marker giantin by confocal microscopy. In control A549 cells, giantin expression extended throughout the entire perinuclear region, whereas in cells expressing exogenous pre-miR-199a or sh-ARHGAP21, giantin showed a highly condensed distribution in part of the perinuclear region (Fig. 6A). When control cells were analyzed in detail using super-resolution microscopy, giantin was closely localized with a *trans*-Golgi marker p230 in the perinuclear region (Fig. 6B), whereas it was in close vicinity to the ER marker PDI not only in preinuclear region but also in the peripheral region (Fig. 6C). In A549 cells transduced with the pre-miR-199a vector, giantin was less associated with PDI, whereas it was mostly present in a condensed area with the *trans*-Golgi marker p230 (Fig. 6B and C). Giantin reportedly resides at the cytoplasmic face of the Golgi membrane and in COPI vesicles and has been shown to function in vesicle tethering at the *cis*-Golgi by associating with p115 and GM130^30^. Accordingly, our observations may suggest that giantin is normally transported between the Golgi and the ER. Upon the high level expression of pre-miR-199a, however, giantin was found to become concentrated in the Golgi region, probably because of reduced Golgi-to-ER retrograde transport. In most ARHGAP21-depleted A549 cells, giantin showed a similar localization to that of pre-miR-199a-expressing cells (Fig. 6A).

Consistent with these observations, abnormal aggregation of Golgi cisternae was frequently observed by TEM analysis of both miR-199a- and sh-ARHGAP21-expressing cells, in contrast to the flattened and stacked Golgi cisternae observed in control cells (Fig. 6D and Supplementary Figs S3 and S4). The cisternae morphology in these aggregates seemed to be swollen, fragmented, or degraded. However, despite the significant effect of miR-199a and sh-ARHGAP21 on the morphology of Golgi cisternae, p230 and giantin did not colocalize (Supplementary Fig. S3), suggesting that the compacted Golgi apparatus manages to maintain the identity of each cisterna.

### ARHGAP21 is required to maintain the *trans*-Golgi during HSV-1 infection

**Figure 7:**
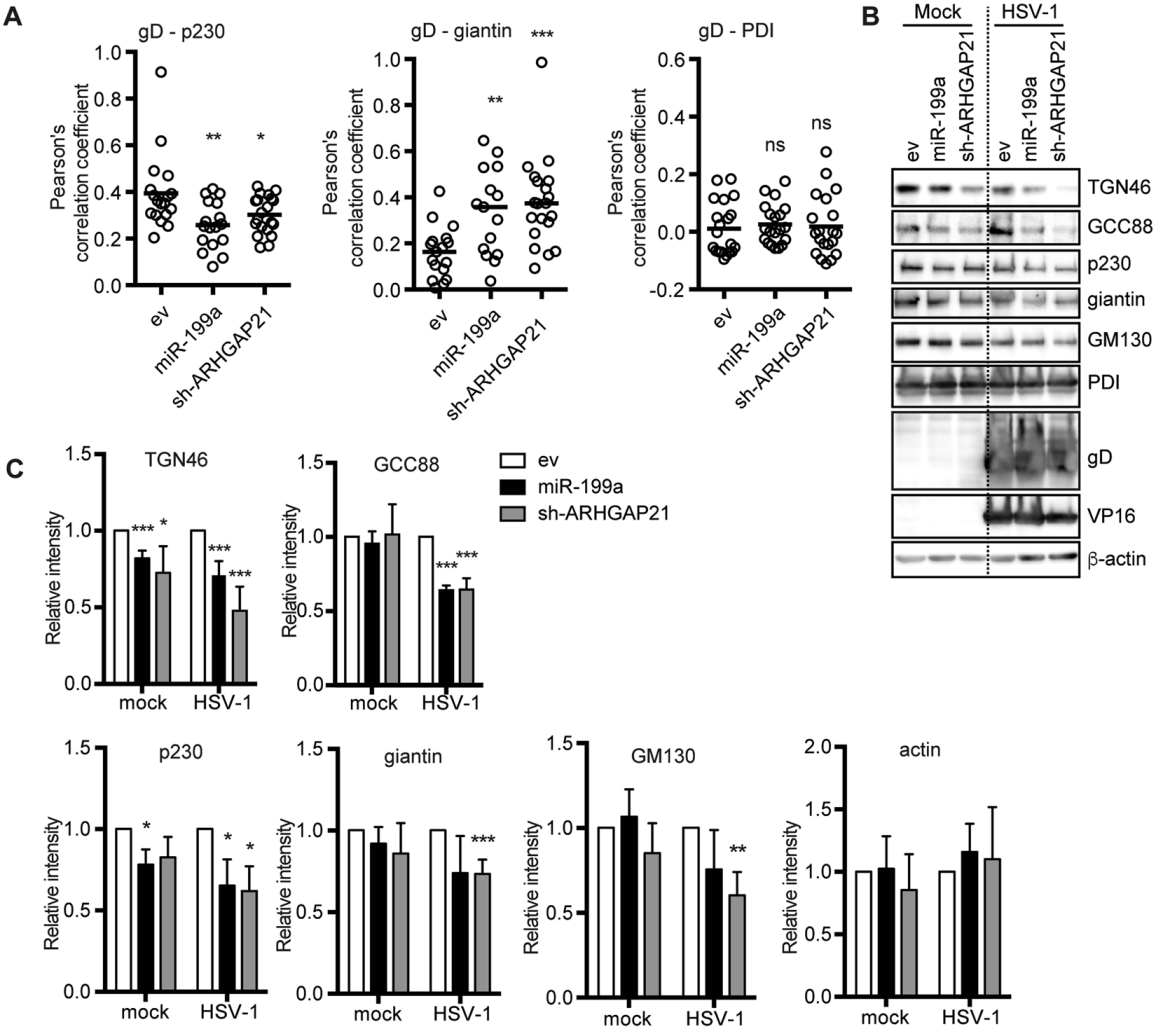
ARHGAP21 is required for maintenance of the *trans*-Golgi during HSV-1 infection. (**A**) To quantify the colocalization of gD with the organelle markers p230 (*trans*-Golgi), giantin (medial- and *cis*-Golgi), and PDI (ER), the Pearson’s correlation coefficient was determined for confocal images of HSV-1-infected A549 cells (moi of 5/12 hpi) transfected with control (ev), pre-miR-199a-, or sh-ARHGAP21#01-expressing lentivirus vector. (**B**,**C**) Western blot analysis of TGN (TGN46), *trans*-Golgi (GCC88 and p230), medial- or *cis*-Golgi (giantin and GM130), and ER (PDI) markers, HSV-1 proteins (gD and VP16), and β-actin in uninfected or HSV-1-infected (moi of 5/20 hpi) A549 cells transfected with control (ev), miR-199a-, or sh-ARHGAP21#01-expressing lentivirus vector. Protein levels of these Golgi markers were quantitated by measuring band intensities of western blot images using Image J software. In B, the bars represent the medians and the asterisks indicate the *p* value (*t*-test) compared with control (ev). ns, not significant. *p < 0.05, **p < 0.01, ***p < 0.005.

We further examined the relationship between the functional and morphological alterations in the Golgi apparatus and the reduced efficiency of HSV-1 secondary envelopment in the *trans*-Golgi, both of which are induced by the high expression of miR-199a. Recent reports have clearly demonstrated the importance of effective and appropriate transport of viral envelope proteins to the membrane compartments for the secondary envelopment^17, 18, 31, 32^ Accordingly, we explored the possibility that the reduced efficiency of the secondary envelopment induced by miR-199a is due to a defect in the Golgi apparatus and subsequent reduction in the intra-Golgi transport of HSV-1 envelope proteins. This transport defect would result in an accumulation of envelope proteins in the *cis*-Golgi or the ER, followed by incomplete glycosylation of viral envelope proteins because glycoprotein maturation occurs within the Golgi apparatus. To examine this possibility, we observed the colocalization of gD with a *trans*-Golgi (p230), medial- and *cis*-Golgi (giantin), or ER (calnexin) marker and performed statistical analysis (Fig. 7A). In miR-199a- and sh-ARHGAP21-expressing cells, the colocalization of gD with p230 was decreased, whereas that of gD and giantin was increased. However, no accumulation of gD in the ER was observed under such conditions. Incomplete glycosylated forms of gD (immature forms), as described previously in Rab1a/b-depleted cells^32^, were not found on western blot analysis to have accumulated in miR-199a- and sh-ARHGAP21-expressing cells (Fig. 7B). These results suggest that miR-199a alters the intra-Golgi distribution of gD without delaying its transport and glycosylation.

From our observations with super-resolution microscopy, we further noticed a reduction in p230 signals, which seemed to be responsible for reduced colocalization of gD with the *trans*-Golgi marker. In fact, our western blotting analysis showed a reduction of Golgi markers in miR-199a- or sh-ARHGAP21-transduced cells, notably TGN and *trans*-Golgi markers (Fig. 7B,C). These reductions were less clear in uninfected cells than in HSV-1 infected cells, suggesting that HSV-1 infection enhances reduction of *trans*-Golgi markers. From these results, we speculate that ARHGAP21 plays an important role in the maintenance of the *trans*-Golgi in HSV-1-infected cells for the efficient secondary envelopment of HSV-1 at the *trans*-Golgi.

## Discussion

To better understand how miR-199a impairs HSV-1 replication, we first examined kinetics of viral gene expression and have shown that miR-199a impaired late stages of HSV-1 replication (Fig. 1 and Supplementary Fig. S1). Further analyses suggested that the secondary envelopment pathway would be one of major targets of this miRNA and also that miR-199a is less likely to impair nuclear egress and secretion of infectious virion to extracellular space, given our results (Fig. 1H and Supplementary Fig. S1). However, these evidences still cannot exclude the possibility that there are additional targets of miR-199a, which includes assembly of tegument on cytoplasmic capsids and transport of envelope proteins to the site of secondary envelopment. We cannot discriminate these steps from secondary envelopment, because suppression of these steps would result in quite similar phenotypes. To identify a target gene of miR-199a, we largely focused on secondary envelopment because techniques to comprehensively analyze tegument assembly and transport of envelope proteins are currently unavailable and because target prediction analysis showed many candidate targets of miR-199a in the Golgi apparatus (Table 1).

To analyze secondary envelopment, we used three imaging techniques: TEM, confocal microscopy, and N-SIM. We conducted TEM analysis to quantitatively observe enveloped and naked capsids and deformation of the Golgi cisternae, confocal microscopic analysis to know the alteration of the Golgi apparatus induced by pre-miR-199a or sh-ARHGAP21 using organelle- and viral-protein specific antibodies at low magnification, and N-SIM analysis to further dissect the association between these proteins at 100-nm level. These analyses complemented each other and indicated consistent results.

Since the cytoplasmic membrane compartment for HSV-1 secondary envelopment is still the subject of debate, we have started to analyze where the secondary envelopment would occur. Our current analyses in A549 cells indicate that the *trans*-Golgi is a potential membrane compartment for secondary envelopment (Fig. 2).

**Figure 8:**
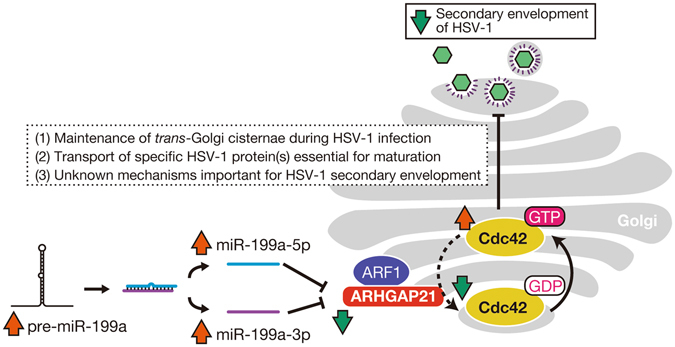
A possible pathway for pre-miR-199a-mediated regulation of HSV-1 secondary envelopment. Dashed lines denote hypothetical mechanisms. Both miR-199a-5p and miR-199a-3p generated from pre-miR-199a inhibit ARHGAP21 protein synthesis, resulting in the accumulation of GTP-binding Cdc42 (activation) in the Golgi apparatus. This activation would disturb secondary envelopment of HSV-1. Disturbance of secondary envelopment may be due to low efficiency in maintenance of *trans*-Golgi cisternae during HSV-1 infection, transport of some HSV-1 protein(s) essential for virion maturation, or unknown mechanism(s).

We demonstrate from our present findings that both miR-199a-5p and -3p down-regulate their direct target *ARHGAP21* (Fig. 3). Importantly, ARHGAP21 is clearly required for efficient secondary envelopment of HSV-1 (Fig. 4) and is localized at the membrane compartment for secondary envelopment in HSV-1-infected A549 cells (Fig. 4D). In addition, Cdc42 activation was shown to reduce the efficiency of secondary envelopment (Fig. 4G). These data strongly suggest that secondary envelopment occurs in the membrane compartment where ARHGAP21 is either naturally localized or efficiently recruited by viral or host factors, and also indicate that ARHGAP21 enhances secondary envelopment probably through the inactivation of Cdc42 (summarized in Fig. 8). Notably and importantly, a previous study has described the importance of ARF1, which recruits ARHGAP21 to the Golgi apparatus, in HSV-1 replication^33^. Together with several previous reports^25, 26^, our current results are consistent with the idea that the ARF1-ARHGAP21-Cdc42 pathway regulated by miR-199a has an impact on HSV-1 replication at the secondary envelopment step, whereas several pieces of evidence are still lacking to completely establish this. For example, miR-199a could suppress the secondary envelopment as well as its immediately preceding steps thorough other target molecules in addition to ARHGAP21. Our previous studies showed that several target genes of miR-199a contribute to the formation of gene regulatory networks observed in type 1 or type 2 cells of epithelial tumor^8, 9^. Indeed, the efficiency of secondary envelopment was clearly different between these two cell types (Fig. 5). Our analysis has also predicted 21 target genes of miR-199a-5p and/or -3p (Table 1) among genes associated with the function of the Golgi apparatus. Therefore, it is possible that some of them could also partly contribute to cellular competency to HSV-1 via miR-199a. Significance of ARHGAP21 as a miR-199a target on this step would be evaluated by experiments testing whether suppression of HSV-1 replication by miR-199a expression can be rescued by the exogenous expression of *ARHGAP21* cDNA. But it was not possible because *ARHGAP21* cDNA is too large (5,877 kb) to be packaged into retro/lenti virus vectors. It would be also interesting to test the impact of exogenous miR-199a expression on the Cdc42 activity in the Golgi apparatus using a Cdc42 biosensor and FRET probes as a future project^34, 35^.

It is still not fully understood how Cdc42 and ARHGAP21 are involved in Golgi function, albeit with some reports demonstrating their function in the Golgi apparatus^25, 27–29^. Therefore, the detailed molecular mechanisms underlying the miR-199a inhibition of HSV-1 secondary envelopment in the *trans*-Golgi remain unresolved. However, we speculate that these molecules would be required for maintenance of *trans*-Golgi upon HSV-1 infection to continuously produce the site for secondary envelopment since the reduction of *trans*-Golgi proteins in miR-199a- and sh-ARHGAP21-expressing cells after HSV-1 infection was observed (Fig. 7C and D).

Another explanation for the contribution of ARHGAP21 to the secondary envelopment of HSV-1 is that the transport efficiency of HSV-1 structural proteins essential for maturation of HSV-1 virion is dependent on ARHGAP21 activity. Our present data indicate that the transport of gD is not affected by knockdown of ARHGAP21 or by exogenous expression of pre-miR-199a, but we cannot exclude the possibility that some other HSV-1 proteins are specifically affected by ARHGAP21.

All of the other viruses whose replication is inhibited by miR-199a, including β and γ herpesviruses, Semliki Forest virus, HCV, and hepatitis B virus^1–3^, acquire an envelope in the cytoplasmic membrane compartment. Notably, it has been already shown that HCV replication is increased by the depletion of Cdc42^36^, suggesting the importance of the miR-199a/ARHGAP21/Cdc42 pathway even for HCV replication. Therefore, it will be important in future studies to determine whether miR-199a can inhibit viruses other than HSV-1.

## Methods

### Cells and viruses

The following human cell lines were used in this study: A549 (non-small-cell lung carcinoma,), NCI-H1299 (non-small-cell lung carcinoma), NCI-H522 (non-small-cell lung carcinoma), HUTU80 (duodenum carcinoma), HEC-1B (endometrial adenocarcinoma), HeLaS3 (cervical carcinoma), M14 (melanoma), and SW13 (adrenocortical carcinoma). All cultures were maintained in Dulbecco’s modified Eagle’s medium containing 10% foetal calf serum. The A549, NCI-H1299, NCI-H522, and M14 cell lines were purchased from American Tissue Culture Collection. HuTu80 and HeLaS3 cell lines were obtained from the Cell Resource Centre for Biomedical Research, Institute of Dvelopment, Aging and Cancer, Tohoku University, Japan. HSV-1 wild-type strain F was propagated in Vero cells, extracted from these infected cells by freeze-thawing and sonication, and purified.

### Antibodies

Anti-ARHGAP21 rabbit IgG, anti-β-actin mouse IgG1 (C4 clone), anti-α-tubulin mouse IgG1 (DM1A clone), anti-ICP0 mouse IgG1 (5H7 clone), anti-ICP4 mouse IgG1 (10F1 clone), anti-ICP8 mouse IgG1 (11E2 clone), anti-gD mouse IgG2a (DL6 clone), anti-VP16 mouse IgG2a (14–5 clone), and anti-VP5 mouse IgG1 (6F10 clone) were purchased from Santa Cruz Biotechnology. Anti-giantin rabbit IgG, anti-PDI mouse IgG2a (RL90 clone), and anti-TGN46 rabbit IgG were purchased from Abcam. Anti-p230 mouse IgG1 (15 clone) and anti-GM130 IgG1 (35 clone) were purchased from BD Biosciences. Anti-FLAG IgG1 (M2) was purchased from Sigma-Aldrich. Anti-GCC88 rabbit IgG (HPA021323) was purchased from Atlas Antibodies. Anti-calnexin rabbit IgG (#2433) was purchased from Cell Signaling.

### Plasmid constructions

The lentivirus vector pLSP-pre-miR-199a plasmid was constructed previously^9^. The pLSP-pre-miR-214 plasmid was generated as previously described^9^ using the primers listed in Supplementary Table S3.

To construct shRNA-expressing vectors, pairs of oligonucleotides encoding gene-specific shRNA were synthesized (Supplementary Table S2) and inserted into the BbsI/EcoRI sites of pmU6. The pmU6-shCre#4 plasmid^37^ was used as the sh-control. These pmU6 plasmids were digested with BamHI and EcoRI and inserted into the BamHI/EcoRI site of pLSP.

The TuD-miR-199a-5p and TuD-miR-199a-3p expression plasmids had been constructed previously^9^. The TuD-miR-214 expression plasmid was constructed according to a previously described protocol^13^ using the oligonucleotides listed in Supplementary Table S2. These plasmids were digested with BamHI and EcoRI and inserted into the BamHI/EcoRI site of pLSP. To construct retrovirus vectors expressing exogenous proteins, a pair of oligonucleotides encoding the FLAG epitope tag was synthesized (Supplementary Table S2) and inserted into the BamHI/EcoRI site of pcDNA3.1(−), designated pcDNA-flag. Protein-coding sequences were amplified by RT-PCR of A549 cDNA using the primers listed in Supplementary Table S3. The amplified fragments were then cloned into the EcoRI/XhoI site of pcDNA-flag.

The retrovirus vector pSC-IP was generated as follows: the BamHI and EcoRI restriction sites in the U3 region located in the 3′-long terminal repeat of pSSSP plasmid^38^ were removed and the region containing the SV40 promoter and the puromycin-resistant gene was replaced by a sequence containing CMV promoter-IRES-puromycin-resistant gene. A multi-cloning site (MCS) from pcDNA3.1(−) was inserted between the CMV promoter and IRES. FLAG-Cdc42 wild type, FLAG-Cdc42 CA mutant, and FLAG-eGFP were inserted into the BamHI/XhoI site in the MCS of pSC-IP.

To construct luciferase reporter plasmids, the 3′UTRs of ARHGAP21 were amplified by RT-PCR of A549 cDNA using the primers listed in Supplementary Table S3 and cloned into the XhoI/NotI site of psiCHECK2 (Promega). Targeted point mutations were generated using a KOD-plus mutagenesis kit (Toyobo) and the primers listed in Supplementary Table S4 in accordance with the manufacturer’s instructions.

### miRNA mimics

miRIDIAN miRNA mimic negative control #1 (NC1), miR-199a-5p, miR-199a-3p, and miR-214 were purchased from Dharmacon.

### Generation and transduction of lentiviral and retroviral vectors for the establishment of stable cell lines

Vesicular stomatitis virus (VSV)-G-pseudotyped lentiviral vectors were produced with the prepackaging cell line HEK-293FT using the ViraPower Lentiviral Expression System (Invitrogen) in accordance with the manufacturer’s instructions. Transductions were then carried out as described previously^13^. VSV-G-pseudotyped, replication-defective, retrovirus vectors were prepared and transduced into cells as described previously^39^. All transduced cells were selected with 1 µg/ml of puromycin from 1 day after the transduction for at least 5 days.

### HSV-1 replication assay

In our current analysis, MOI was defined as the ratio of titre (pfu) of HSV-1 determined in Vero cells to the number of cells inoculated, because HSV-1 forms almost the same number of plaques in Vero cells and A549 cells but plaques formed in Vero cells are much clearer and much suitable for statistically accurate titration. Cells were inoculated with viral stock at an moi of 0.01 or 5 and incubated for 1 h at 37 °C. The virus solution was aspirated and replaced with new culture medium. At 24, 36, 48, or 72 h after inoculation, supernatants and/or infected cells were collected for analyses of virus titres, protein, and RNA. Cell-associated viruses were extracted from the infected cells by three cycles of freeze-thawing and sonication. The titres of the collected viruses were determined using a plaque assay with Vero cells^40^.

### Quantitative PCR

Extraction of total RNA and quantification of mRNA or miRNA were performed as described previously^8^ using the primers listed in Supplementary Table S5.

### Western blotting

Western blotting was performed as described previously^41^.

### Dual luciferase assay

Cells were seeded at a density of 1 × 10^5^ cells per well in 24-well plates in DMEM containing 10% FBS the day before transfection and then transfected in triplicate with Lipofectamine 2000 (Invitrogen) and 100 ng of psiCHECK2 reporter plasmid and 25 nM miRNA mimic. At 48 h after transfection, a dual-luciferase assay was performed using Dual-Luciferase Reporter Assay System (Promega) and read on a Glomax luminometer (Promega) in accordance with the manufacturer’s instructions.

### Immunofluorescent assay

For staining HSV-1-infected and -uninfected cells, cells were seeded in 8-well chamber slides the day before infection and inoculated with HSV-1 at an moi of 5. At 12 h after the inoculation, cells were fixed with 4% paraformaldehyde at 37 °C for 10 min. After being washed twice with PBS, the fixed cells were incubated with blocking buffer consisting of 2.5% BSA, 50% Blocking One solution (Nacali Tesque), 0.01% sodium azide, and 0.1% saponin in PBS for 1 h at room temperature and then a combination of antibodies diluted in the blocking buffer overnight at 4 °C. After three washes with PBS containing 0.1% saponin, the cells were incubated with 2 µg/ml Alexa Fluor 488 anti-rabbit IgG, Alexa Fluor 555 anti-mouse IgG2a, Alexa Fluor 647 anti-mouse IgG1 (Invitrogen), and/or Acti-stain 555 (Cytoskeleton) diluted in the blocking buffer for 1 h at 37 °C. After three further washes with PBS containing 0.1% saponin, the slides were mounted with coverslips using Vectashield with DAPI (Vector Laboratory). Images were acquired with a confocal microscope (Nikon A1) and/or a super-resolution microscope (Nikon N-SIM). To evaluate colocalization, the acquired images were analyzed using the ImageJ plug-in Colocalization Threshold.

### Transmissible electron microscopy

HSV-1-infected cells at an moi of 5 for 20 h were fixed with 2.5% glutaraldehyde in 0.1 M sodium phosphate buffer (pH 7.4) for 2 h at room temperature. After fixation, the cells were harvested and pelleted by centrifugation at 12,000 rpm for 5 min. The samples were then washed with the same buffer containing 3% sucrose, postfixed with 1% osmium tetroxide in the same buffer for 2 h on ice, dehydrated with a graded ethanol series followed by propylene oxide, and embedded in Epon 812 resin mixture. Ultrathin sections (60-nm-thick) were stained with 2% uranyl acetate in 70% ethanol for 5 min at room temperature and lead citrate for 5 min at room temperature. Images were acquired with a Hitachi H7500 electron microscope.

For TEM analysis of each cell types, we acquired TEM images of almost the entire cells before counting capsids and used all the acquired images (at least 10 images) that are tack sharp and contain clear capsids for the analysis. These images were used to determine the ratio of capsids in the cytoplasm to those in the nucleus and also the ratio of enveloped capsids in cytoplasm. Capsids were here defined as approximately 100 nm particles with sharply bordered rim. Using these criteria, no capsids were detected in uninfected cells. To quantitate the ratio of capsids in cytoplasm/nucleus, we first measured the area (μm^2^) of both the entire cells (X) and of the nuclei (Y) using Image J software and then calculated the area of cytoplasm (X-Y). After counting capsids in both nucleus and cytoplasm, we calculated the number of capsids in 100 μm^2^ of nucleus and cytoplasm, respectively, then determined the ratios between them.

### Target prediction and Gene Ontology analysis

Using TargetScan release 7.0 (http://www.targetscan.org/) and PicTar (http://pictar.mdc-berlin.de/), the target genes of miR-199a-5p and miR-199a-3p predicted by both algorithms were acquired to create a gene set (84 genes for miR-199a-5p and 53 genes for miR-199a-3p). We further selected genes associated with Golgi function using Gene Ontology terms (21 genes listed in Table 1).

### Statistical analysis

For each representative image, gel, immunoblot, or graph, the experiments were independently repeated at least twice and there were no limitations in repeatability. GraphPad PRISM version 6.0f (GraphPad Software) was used for the analysis of statistical significance. In the time course experiments, two-way ANOVA was used to evaluate significant differences between an experimental group and the control group. In Fig. 6D, the Mann-Whitney test was used to evaluate significant differences in envelopment efficiency. In other experiments, an unpaired *t*-test was used.

## Electronic supplementary material

Supplementary Information
