# Supplementary material for: MiR-199a Inhibits Secondary Envelopment of Herpes Simplex Virus-1 Through the Downregulation of Cdc42-specific GTPase Activating Protein Localized in Golgi Apparatus
Source: Sci Rep. 2017 Jul 27;7:6650. doi: 10.1038/s41598-017-06754-3 (PMC5532371; doi:10.1038/s41598-017-06754-3)
Supplement: Supplementary file 1 — Supplementary Information [file 41598_2017_6754_MOESM1_ESM.pdf]

Supplementary Information

MiR-199a Inhibits Secondary Envelopment of Herpes Simplex Virus-1 Through the Downregulation of Cdc42-specific GTPase Activating Protein Localized in Golgi Apparatus

Kyousuke Kobayashi, Fumiko Suemasa, Hiroshi Sagara, Shinya Nakamura, Yasushi Ino, Kazuyoshi Kobayashi, Hiroaki Hiramatsu, Takeshi Haraguchi, Kazuo Kurokawa, Tomoki Todo, Akihiko Nakano, and Hideo Iba

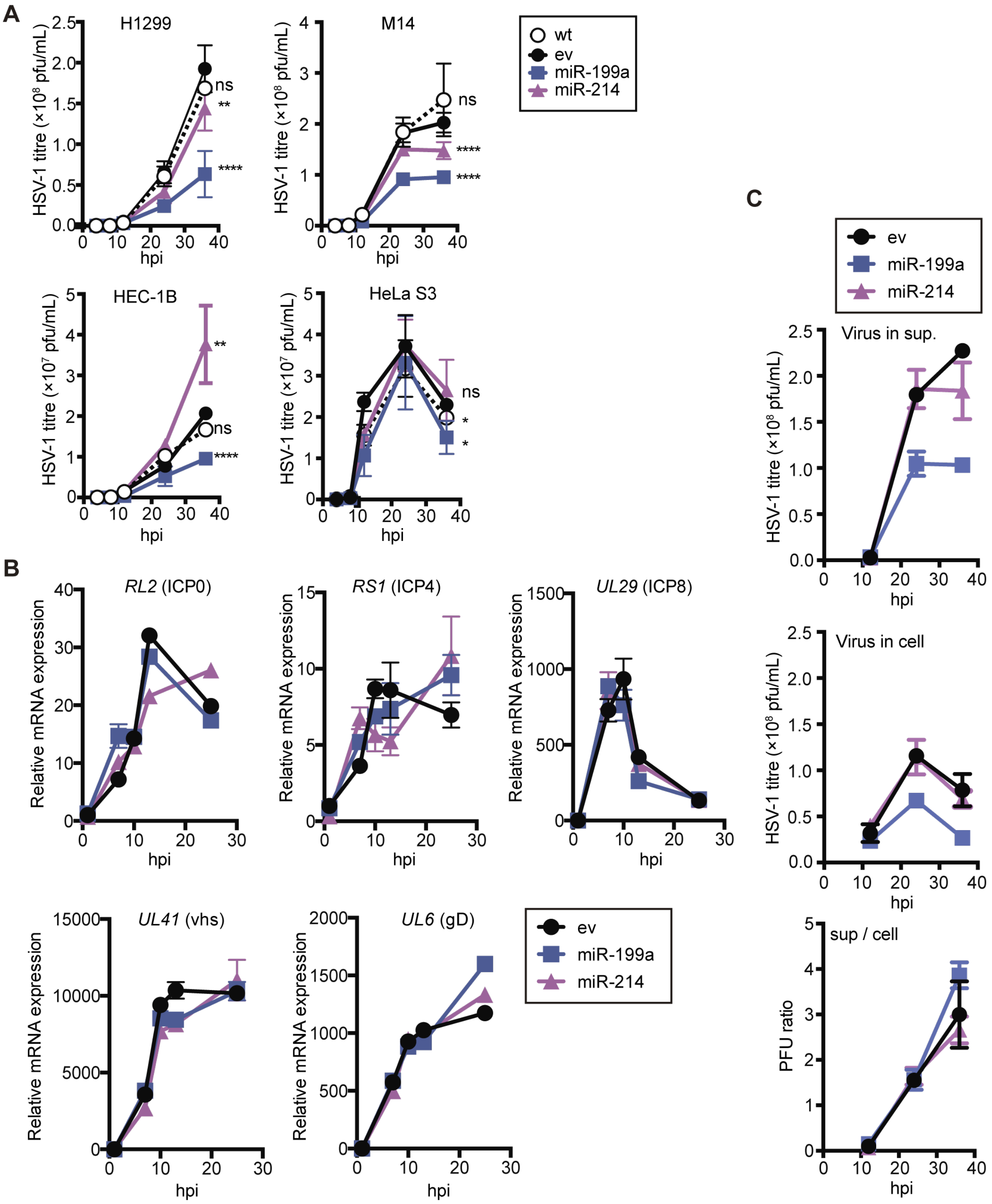

Supplementary Figure S1 MiR-199a-5p and miR-199a-3p inhibit HSV-1 replication in various human cell lines. (A) Four human cell lines (H1299, M14, HEC-1B, and HeLa S3)

transfected with control (ev) and pre-miRNA-expressing lentivirus vector were infected with HSV-1 at an moi of 5. HSV-1 titres in the cell culture supernatant and the extracts from the cells were determined by plaque assay at 4, 8,12, 24, and 36 hpi. (B) HSV-1 mRNA analysis by qRT-PCR analysis. A549 cells transfected with control (ev) and pre-miRNA-expressing lentivirus vector were infected with HSV-1 at an moi of 5 and total RNA was extracted for the quantification of immediate early (*RL2* [ICP0] and *RS1* [ICP4]), early (*UL29* [ICP8]), and late (*UL41* [vhs] and *US6* [gD]) genes at 1, 7, 10, 13, 25 hpi. 18S ribosomal RNA was used as an internal control. The data represent the means ± s.d. (n=3). (C) The HSV-1 titer of supernatant and of infected cells and the ratio between them in HSV-1 infected (moi of 5) A549 cells transduced with a control (EV) or a pre-miRNA-expressing lentivirus vector at 12, 24, and 36 hpi. The asterisks indicate the p value (two-way ANOVA) compared with control (ev). ns, not significant. *$p<0.05$, **$p<0.01$, ****$p<0.001$.

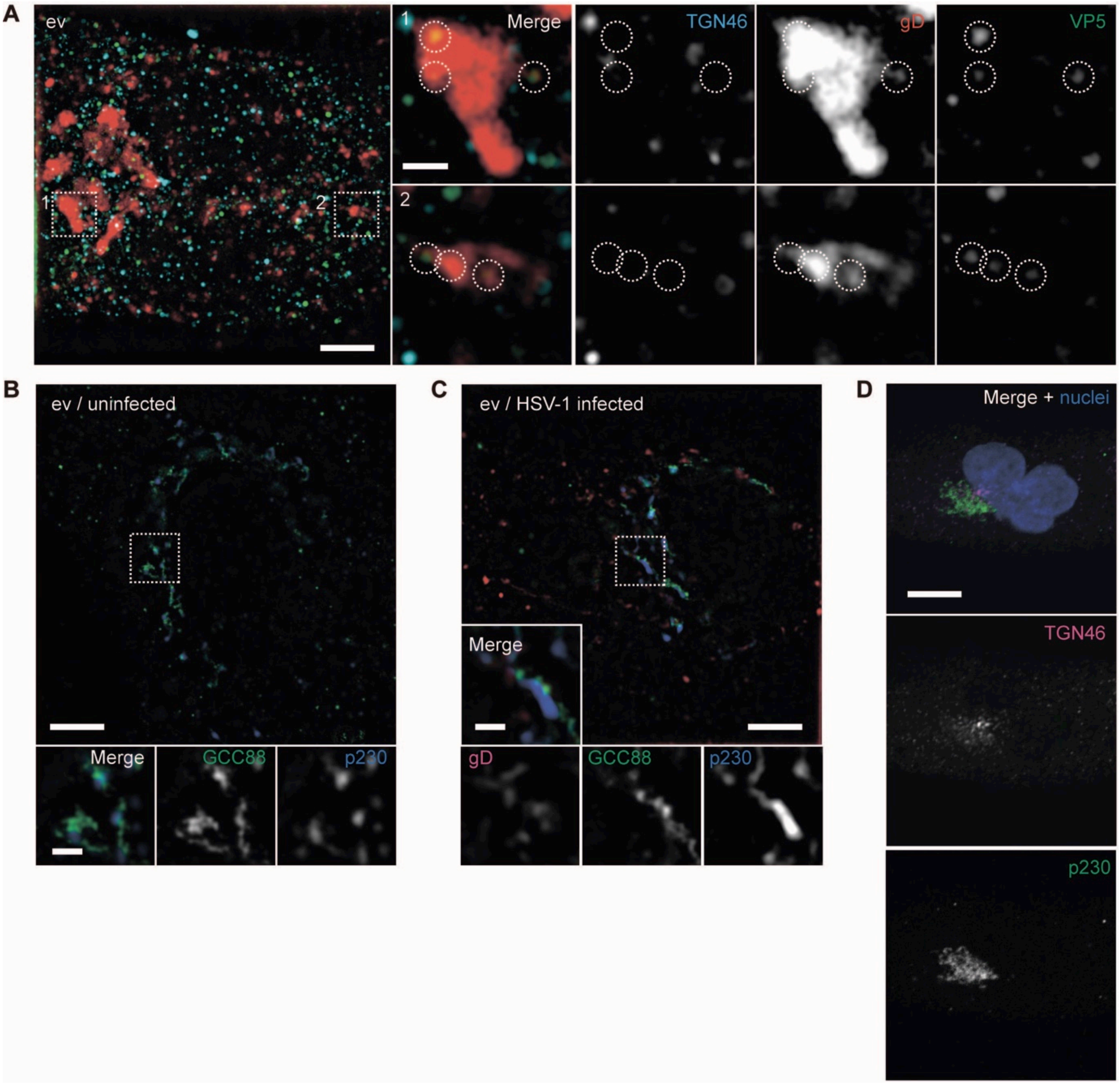

Supplementary Figure S2 Localization of TGN and *trans*-Golgi markers in HSV-1-infected or uninfected A549 cells. (A) Representative super-resolution images of HSV-1-infected A549 cells (moi of 5/12 hpi) transfected with control (ev) lentivirus vector. Cells were stained with anti-gD antibody (red), anti-VP5 antibody (green), and anti-TGN46 antibody (cyan). The furthest left panel shows a z-stack image reconstructed by maximum

intensity projection (bars, 5 µm) and the other panels show magnifications of the two areas boxed with dashed lines (1,2) in the left panel (bars, 1 µm). The circles indicate capsids associated with or included in gD-positive membrane compartments. (B and C) Representative super-resolution images of uninfected A549 (B) or HSV-1-infected A549 cells (C) (moi of 5/12 hpi) transfected with control (EV) lentivirus vector. Cells were stained with anti-GCC88 antibody (green) and anti-p230 antibody (blue). In HSV-1-infected cells, gD was also stained (C, red). The upper panels show z-stack images reconstructed by maximum intensity projection (bars, 5 µm) and the lower panels show magnifications of the areas boxed with dashed lines in the upper panels (bars, 1 µm). (D) Representative confocal microscopy images of A549 cells stained with anti-TGN46 antibody (magenta), anti-p230 antibody (green), and DAPI (blue) (bars, 5 µm).

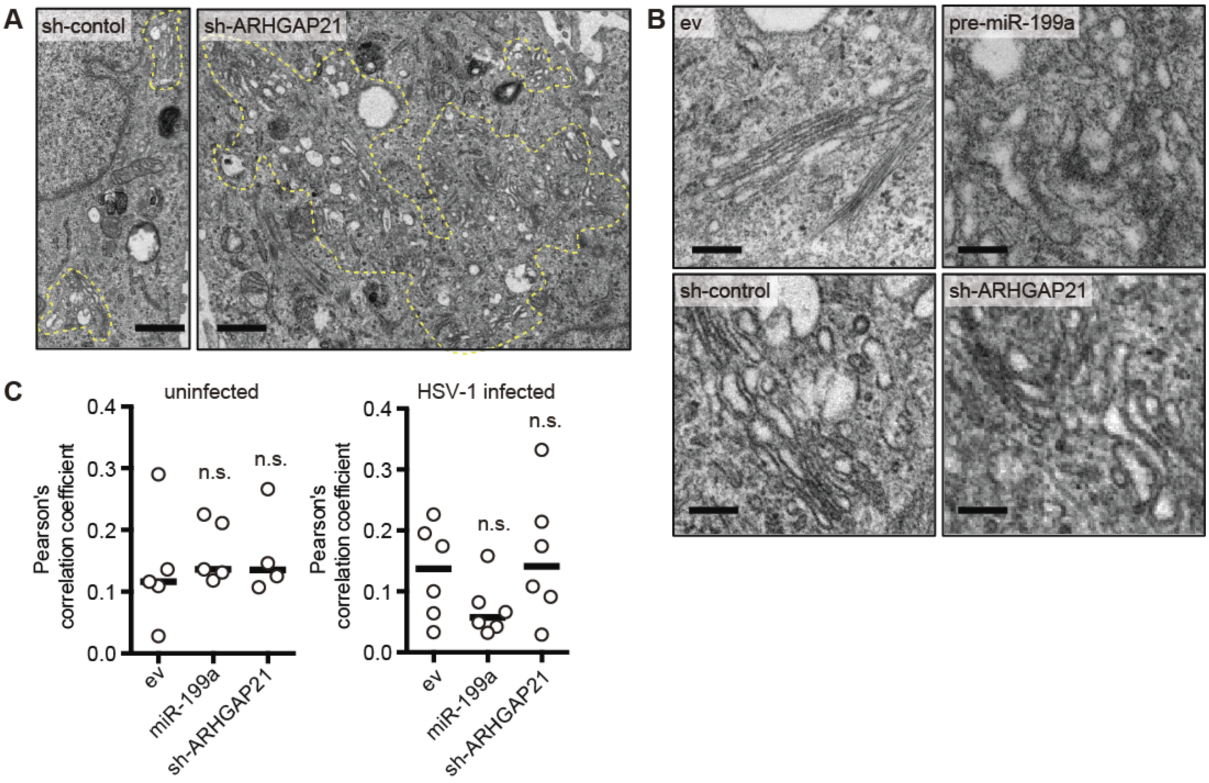

Supplementary Figure S3 MiR-199a alters the morphology of the Golgi cisternae without affecting their identity. (A and B) Representative transmission electron micrographs of A549 cells transduced with sh-control-, sh-ARHGAP21#01-, control (ev)-, and pre-miR-199a-expressing lentivirus vector (bars in A, 1 µm; bars in B, 200 nm). In A, the areas enclosed by the dashed yellow lines indicate the Golgi apparatus. (C) Quantification of the colocalization of p230 (*trans*-Golgi) with giantin (medial- and *cis*-Golgi). Using super-resolution images of uninfected or HSV-1-infected (moi of 5/12 hpi) A549 cells transfected with control (ev), pre-miR-199a-, or sh-ARHGAP21#01-expressing lentivirus vector, Pearson's correlation coefficients were determined. In C, the bars represent the

medians and n.s. indicates no significant difference compared with control (ev) by *t*-test.

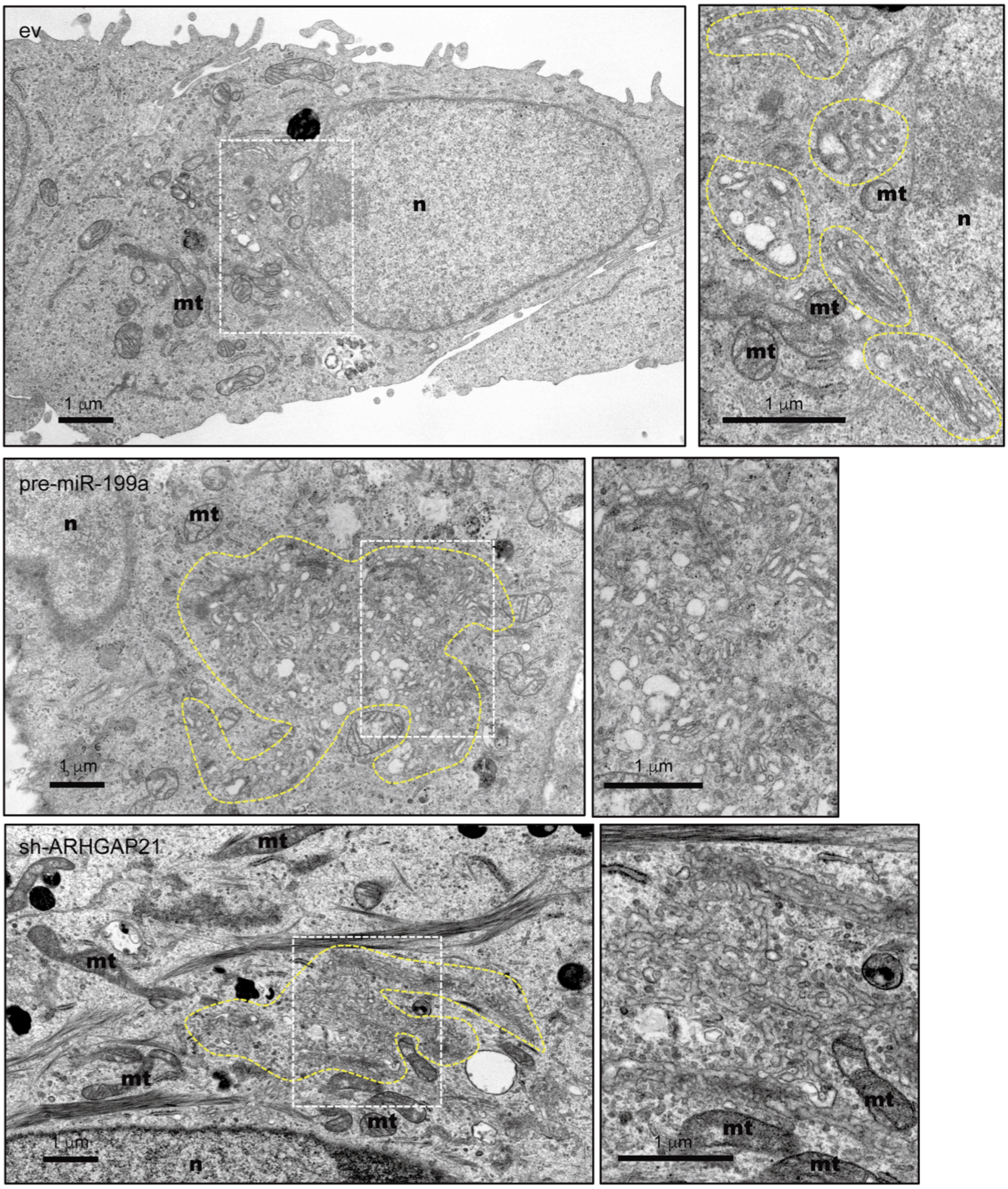

Supplementary Figure S4 Additional TEM images of the Golgi apparatus.

Representative transmission electron micrographs of A549 cells transduced with control (ev)-, and pre-miR-199a,- sh-ARHGAP21#01-expressing lentivirus vector. the areas enclosed by

the dashed yellow lines indicate the Golgi apparatus. The right panels show magnifications of the areas boxed with white dashed lines in the left panels. n, nucleus; mt, mitochondria.

Supplementary Table S1 Summary of TEM analysis

Cells		No. of enveloped capsids in cytoplasm (%)	No. of non-enveloped capsids in cytoplasm (%)	No. of analyzed cells*
A549	ev	519 (80.3)	127 (19.7)	15
	pre-miR-199a	332 (59.4)	227 (40.6)	16
	sh-control	177 (74.4)	61 (25.6)	10
	sh-ARHGAP21 #1	104 (35.1)	192 (64.9)	18
	sh-ARHGAP21 #2	38 (29.0)	93 (71.0)	11
	sh-ARHGAP21 #3	71 (58.7)	50 (41.3)	12
	control	116 (67.4)	56 (32.6)	11
	Cdc42 WT	82 (64.1)	46 (35.9)	10
	Cdc42 CA	57 (41.3)	81 (58.7)	12
H522		43 (33.6)	85 (66.4)	10
SW13		141 (34.9)	263 (65.1)	11
H1299		159 (53.4)	139 (46.6)	11
HeLa S3		84 (53.2)	74 (46.8)	10
AZ521		109 (18.9)	468 (81.1)	10
A549		253 (59.8)	170 (40.2)	10

* 2 – 4 independent cellular samples were analyzed.

Supplementary Table S2 List of oligonucleotides used to construct shRNA, TuD RNA, and an epitope tag.

shRNA and TuD	Sense	Antisense
sh-ARHGAP21 #01	tttgaaagattagatagtttaagagcttcctgtcactcttaaactatctaatctttcttttttg	aattcaaaaaagaaagattagatagtttaagagtgacaggaagctcttaaactatctaatcttt
sh-ARHGAP21 #02	tttgataacttacttacattgaatgcttcctgtcacattcaatgtaagtaagttatcttttttg	aattcaaaaaagataacttacttacattgaatgtgacaggaagcattcaatgtaagtaagttat
sh-ARHGAP21 #03	tttggatttgtcccttctttgtcagcttcctgtcactgacaaagaagggacaaatccttttttg	aattcaaaaaaggatttgtcccttctttgtcagtgacaggaagctgacaaagaagggacaaatc
TuD-miR-214	catcaacactgcctgtctgcttctgcctgctgtcaagtattctggtcacagaatacaacactgcctgtctgcttctgcctgctgtcaag	tcatcttgacagcaggcagaagcagacaggcagtgttgtattctgtgaccagaatacttgacagcaggcagaagcagacaggcagtgtt
FLAG tag	gatccgccaccatggactacaaagacgatgacgacaagg	aattccttgtcgtcatcgtctttgtagtccatggtggcg

Supplementary Table S3 List of primer pairs used for PCR cloning.

PCR	Forward	Reverse
pre-miR-214	gaagactgtttggaaacctgaaggaaccaagggcctggctggacag agttgtcatgtgtctgcctgtctacacttgctgtgcagaacatccgctca cctgtac	gaattcagggctgctttctttcaatggctggttgtcattcaggctgggtt gtcatgtgactgcctgtctgtgcctgctgtacaggtgagcggatgttc
CDC42 (CDS)	cggaattcatgcagacaattaagtgtgttgttgtg	ccgctcgagtcatagcagcacacacctgc
eGFP (CDS)	cgggatccgtgagcaagggcgaggagc	ccgctcgagcttgtacagctcgtccatgccg
ARHGAP21 (3'UTR)	attctaggcgatcgcactgggggtatgtccactctagc	tttattgcggccagctccagtgtttaattgggtatgcacacag

Supplementary Table S4 List of primer pairs used for site-directed point mutagenesis.

mutagenesis	Forward	Reverse
Cdc42 CA (Q61L)	ctagaggattatgacagattacgaccg	ccctgcagtatcaaaaagtccaag
ARHGAP21 (3’UTR) 3p mut	ataagccagaagaacatatatatatata	gtaatgcactgtaaagctatttcac
ARHGAP21 (3’UTR) 5p mut	agctgagctggccgcaataaaatatc	gtttaattgggtatgcacacagg

Supplementary Table S5 List of primer pairs used for qPCR.

qPCR	Forward	Reverse
ARHGAP21	catctgaagacagtggcaga	agtcatggtgctggatgagc
GAPDH	ctctgctcctcctgttcgac	ttaaaagcagccctggtgac
RNA18S5	gaaacttaaaggaattgacggaagg	ggtgaggtttcccgtg
ICP0 (*RL2*)	cgtgaacaagacgatcacgg	atgtttcccgtctggtccac
ICP4 (*RS1*)	gtcgatgcttgggtgggaaa	gccagagacagaccgtcaga
ICP8 (*UL29*)	gttaccttgtccgagcctcc	cgacctcaaacacgagacga
vhs (*UL41*)	gggaatgtagcaggcgctaa	tgcgccaacctctatcacac
gD (*US6*)	cgtccggaaacaaccctaca	ccaggttatcctcgctgac
